# Identifiable biomarker and treatment development using HIV-1 long term non-progressor sera

**DOI:** 10.1186/s12865-015-0094-z

**Published:** 2015-04-28

**Authors:** Yuxia Hao, Ge Bai, Junping Wang, Longfeng Zhao, Kyle Sutherland, Jianfeng Cai, Chuanhai Cao

**Affiliations:** Shanxi Provincial People’s Hospital, Shanxi, China; Department of Chemistry, University of South Florida, Tampa, FL USA; Shanxi Medical University, Shanxi, China; Department of Pharmaceutical Sciences, College of Pharmacy, University of South Florida, Tampa, FL USA; USF-Health Byrd Alzheimer’s Institute, University of South Florida, 4001 E. Fletcher Ave, Tampa, FL 33613 USA

**Keywords:** HIV, AIDS, LTNP, Long-term non-progressor, Monoclonal antibody, Epitope, Virus

## Abstract

**Background:**

HIV-infected long-term non-progressor (LTNP) subjects can prevent viral replication and may harbor useful information for the development of both antibody and active vaccination treatments. In this study we used LTNP sera to examine the epitopes presented to the gp160 protein, and from this procedure we hope to elucidate potential biomarkers pertaining to the level of resistance a patient may have in developing AIDS after infection with HIV. We used five clinical sera samples from LTNP patients to identify common epitopes by ELISA; peptides with high binding to sera were selected and analyzed for conservation among HIV clades. Antibodies were generated against one identified epitope using a chimeric peptide in BALB/c mice, and both the sera from these mice and LTNP sera were tested for viral inhibition capabilities.

**Results:**

A monoclonal antibody, CL3, against one identified epitope was used to compare these epitopes neutralizing capability. LTNP sera was also studied to determine chemokine/cytokine changes in these patients. The sera from LTNP patients 2, 3, 4, and 5 were identified as having the highest titers, and also significantly inhibited syncytia formation in vitro. Finally, the protein cytokine array demonstrated that I-309 and IGFBP-1 decreased in LTNPs, but levels of TIMP-1 and NAP-2 increased significantly.

**Conclusions:**

Our results indicate that the use of LTNP samples may be a useful for identifying further anti-viral epitopes, and may be a possible predictor for determining if patients show higher resistances of converting the HIV infection to AIDS.

## Background

Cell-mediated immunity (CMI) is important in the control of the immune system and its downstream effects, and plays a vital role in the elimination of viral infections [1]. In infections pertaining to the human immunodeficiency virus (HIV), the humoral response has also been shown to play a distinct role in preventing and containing infections [2]. As branches of the immune system, both cellular and humoral, play a critical role in the management of HIV and viral infections, it is important that potential vaccinations or treatments mimic these effects.

In the past, passive antibody therapy has served as a strategy for the prevention and treatment of various diseases [3-7]. *In vivo* studies have demonstrated the ability of passive immunotherapy to neutralize HIV or SHIV (Simian-human immunodeficiency virus) in animal models [8-14]. Thus, intensive efforts have been made to generate and characterize novel, efficacious neutralizing anti-HIV human antibodies [15,16]. Only a few anti-HIV-1 human monoclonal antibodies (HuMAbs) have been shown to neutralize clinical HIV-1 isolates from HIV infected human B cells. These include b12 and F105, which are directed against the CD4-binding domain of gp120 [17-19], 2G12, which binds to a conserved epitope on the gp120 envelope protein [20,21], and 2F5 & 4E10, which are directed to a highly conserved region of the transmembrane gp41 outside the immunodominant region [22-24].

Several groups have demonstrated that use of a single antibody is not likely to have a clinically significant prophylactic or therapeutic effect against HIV-1 [25-28]. As such, multi-component antibody therapies (e.g. “cocktails”) have been proposed as a more effective alternative, due primarily to their ability to target multiple neutralization epitopes. Development of novel anti-HIV antibodies with broad neutralizing activities may have considerable prophylactic and therapeutic potential as component parts of a “cocktail” preparation. One such HuMAb combination, consisting of 2F5 and 2G12, was tested in a phase I clinical trial and showed a significant decrease in viral loads in several participants [29,30].

With the success of HAART (highly active antiretroviral therapy), HIV-infected subjects can now live for decades longer than what was previously thought. However, at this point there is still no known “cure-all” vaccine that can prevent and treat HIV infection. There is likewise no known antibodies can completely stop viral replication in HIV-infected subjects. For many years, scientists have tried to identify novel epitopes within the HIV peptides, and to develop some combination of an antibody cocktail relating to many diverse epitopes of HIV to help prevent HIV replication.

In recent years, there have, unfortunately, been few breakthroughs towards further development of antibody therapy and vaccine development against HIV/AIDS. In order to design a novel method for the identification of conserved HIV epitopes, we recruited 5 HIV-infected long-term non-progressor (LTNP) subjects. LTNP subjects are patients infected with HIV but have some capabilities of controlling the virus without anti-retroviral therapy. LTNP patients harbor a great deal of useful information for the development of vaccines/antibody treatments, as these patients naturally control infections. In one study, published in 2006, substantially higher levels of 2G12-like antibodies were found in LTNP patients than in the control group, suggesting a higher humoral response in these patients when looking at the HIV-1 envelope epitope [31]. Herein, we report the development and use of an epitope mapping method using LTNP sera for future novel antibody identification, and this method is carried out on the gp160 protein product of the HIV envelope gene (env). Through this procedure, we were able to identify and documents previously discovered epitope, as well as novel epitopes in this region. The lab also studied the neutralization of a clinical viral isolate of HIV-1 by the CL3 antibody, generated in a HIV-1 infected long-term non-progressor (LTNP) subject. CL3 is a monoclonal antibody (designated clone 3) which was found in a previous research study to recognized 10 amino acids (GCSGKLICTT) within the immunodominant region (cluster I) of the transmembrane envelope glycoprotein gp41, and was able to neutralize viral infection to target cells [32]. Besides epitope identification, we also used a protein array assay to find novel protein changes (chemokine, cytokine, etc.) in LTNP subjects when compared to the control. We believe such methods could be the most effective and most beneficial approach in finding novel epitope and anti-viral factors. Those factors identified from LTNPs can be used to generate a treatment cocktail, or add antiviral antibodies to existing drug cocktails. This may also be used as an evaluation tool regarding the level of resistance a particular patient might have as to the timeline regarding the progress of AIDS development.

## Methods

### Ethics Statement

Long term HIV-1 infected LTNPs with elevated anti-HIV antibody titers were used as blood plasma and cell donors. All LTNP subjects had been infected with HIV-1 clade B for over 15 years without progression to AIDS. The University of South Florida’s (USF) Institutional Review Board (IRB) on human research, in accordance with the ethical principles set forth by the World Medical Association (WMA) and the Declaration of Helsinki, and USF’s Institutional Animal Care and Use Committee (IACUC) approved all protocols and methods associated with this study, and all patients studied in the manuscript gave written informed consent for participation in the study.

### Blood collection

Long term HIV-1 infected LTNPs with elevated anti-HIV antibody titers were used as blood plasma and cell donors. All LTNP subjects had been infected with HIV-1 clade B for over 15 years without progression to AIDS.

### Antibody binding analysis (epitope mapping)

HIV-1 MN gp160 peptides (obtained from the AIDS Research and Reference Reagent Program) containing overlapping amino acids were used for ELISA assays as follows. 96 well plates (Immulon II, Dynatech) were coated with different overlapping gp41 peptides and incubated for 60 min at 37°C. The LTNP patient sera were diluted in serial dilutions and incubated for 60 min at 37°C. After washing, a mouse anti human immunoglobulin (IgG or IgM) conjugated to horseradish peroxidase (HRP) was added. HIV-1 negative (normal human sera) controls were used in all plates. Following incubation and washing, TMB substrate was added to the wells as described above. Following color development, the reaction was quantified at 450 nm. Wells with a reading at least three times higher than the background reading in the last two dilutions (i.e. the two diluted sample) were considered positive for binding.

### Identification of the consensus sequence of each epitope by “blast” function

Each of the peptide sequences was copied and put into the sequence editor module of DNAStar. A search for homology was performed by BLAST function. The homology percentage among amino acids was obtained.

### Analysis of CL3 Epitope Homology

HIV gp41 amino acid sequences were analyzed from the GenBank databank, and available gp41 sequences were analyzed with “MegAlign” using DNAStar software. The amino acid sequence GCSGKLIC was used as a standard, and each “MegAlign” sequence was compared to this. Identical or different amino acids were recorded at each residue, followed by sequence comparison analysis. Results were represented as percent homology.

### Peptide vaccine design and vaccination

A chimeric peptide with three CL3 epitopes linked by GKGKGK was synthesized and mixed with an MPL adjuvant. The resulting mixture was injected into three different BALB/c mice. Blood was collected 10 days after injection. The antibody titer was measured with an ELISA assay using HIV gp41 recombinant proteins.

### Syncytia forming inhibition assay

Sera from LTNP patients and mice injected with the above chimeric peptide was diluted at different serial dilutions and mixed with H9MN. After a 30-minute incubation period, they were added to MT2 cells. After a 24 hours incubation period, the number of syncytia was determined using light microscopy.

### Antibodies used in neutralization profiles

CL3 was purified using Protein A agarose beads (Invitrogen, CA). 2F5 and 2G12 were generated and purified by Polymun (Vienna, Austria). Human IgG (designated Cont IgG, purchased from Sigma Chemical Co (St. Louis, MO) was used as a negative control. An additional non-HIV-1 neutralizing HuMAb, designated ContHuMAb (control HuMAb), was also tested as a control reagent.

### Neutralization of Clinical Isolates

The virus stock of HIV-1 was expanded in phytohemagglutinin (PHA) -stimulated peripheral blood mononuclear cells (PBMCs). For all experiments, viruses were passaged in vitro only once from the original virus stocks to maintain previously established phenotypes. PBMCs used for all neutralization assays were either isolated from HIV-uninfected donors by Ficoll-Hypaque centrifugation, or from (for assays performed at the University of South Florida) Leukopaks, whole blood of individual donors purchased from Florida Blood Services (St. Petersburg. FL).

Neutralization assays utilized a virus from the panel of clinical clade B HIV-1 isolates upon recommendation by the National Institutes of Health (NIH), Division of AIDS, and others [18]. These assays were performed with modifications according to the protocol of Zolla-Pazner and Sharpe [33].

Briefly, antibodies were tested starting at 50 μg/mL (50 μl/ well) in triplicates, with subsequent analysis of four-fold dilutions. Fifty microliters of virus at 1000 TCID50/ml were added to each well. The mixture was incubated for 1 hour at 37°C in a CO2 (5%) incubator. After incubation, 106 PHA-stimulated and polybrene-treated PBMCs in 100 (l assay medium containing 20% fetal bovine serum (FBS) were added to each antibody/virus mixture and incubated for 2.5 hours at 37°C. After washing twice, the cells were suspended in culture media containing 10 U/ml IL-2, and incubated at 37°C for 6–9 days. Samples were then lysed by the addition of 20 (l 10% Triton X-100 in phosphate-buffered saline (PBS) per well and incubated at 37°C for 1 hour. HIV-1 p24 concentrations in each well were measured as described.

### Cytokine and Chemokine Array of LTNP Samples

In order to determine which cell-mediated factors played a role in LTNP survival, a cytokine array of T-cells from LTNP patients was conducted. The protein assay was conducted using RayBio Assay Human Cytokine Array, strictly following the manufacturer’s protocol. In short, array membranes were blocked in blocking buffer at room temperature for 30 minutes. After washing, samples of serum from patients (1:10 diluted) were incubated with a membrane at room temperature for 1 to 2 hours. After another wash, the membrane was incubated with a primary biotin-Ab, followed by washing and exposure to HRP-streptavidin as the secondary antibody. The membrane was subsequently washed and developed with ECL substrate followed by a chemiluminescence imaging system. The results were quantified using volume array analysis in Gel analysis software.

### Statistical Analysis

For the ELISA and vaccine portions, data are expressed as the mean ± SD and analyzed using a one-way analysis of variance (ANOVA), and post hoc analysis between groups was analyzed with Fisher’s LSD test. The level of statistical significance was deemed to be P < 0.05.

## Results

### LTNP sera shows high antibody binding to recombinant HIV-1 gp 160 protein

**Figure 1:**
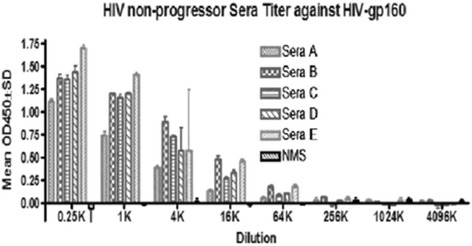
LTNP Sera Analysis. Sera from five LTNP subjects (LTNP-1, LTNP-2, LTNP-3, etc.) and normal serum (NMS) were tested against HIV-1 gp160 protein using an ELISA assay. All 5 subjects had been infected with HIV-1 clade B for over 15 years without developing into AIDS. All subjects showed considerable antibody binding at the endpoint titer, with subjects LTNP-2 and LTNP-5 (patient 25) having the highest antibody end point titers.

Sera from five LTNP subjects (LTNP-1, LTNP-2, LTNP-3, LTNP-4, and LTNP-5) and normal serum (NMS) were tested against the entire peptide library of HIV-1 MN Env gp160 protein using an ELISA assay. All subjects showed antibody binding to the gp41 portion of the gp160 protein, and sera from LTNP-2 and LTNP-3 had the highest antibody end point titers (Figure 1).

### LTNP sera samples epitope mapping and titer detection

After testing the sera against the whole peptide library of HIV-1 MN Env protein, we successfully mapped peptides with high binding to those sera. Seven epitopes were successfully mapped. The sequences and the homology results of a blast search are listed here: Epitope A: QARLLLSGIVQQQMNLLRAT, 100% identical for 100 out of 100 sequences; Epitope B: GCSGKLICTTTVPWNASWSNKSL, range from 95.7-100%, 20 out of 100 are 100%, and 75 out of 100 are 95.7%, however the core epitope KLIC is 100% identical; Epitope C: LLELDKWASLWNWFDITNWLW, 100%, 100 out of 100 sequences, Epitope D: DITNWLWYIKI 100%, 100 out of 100 sequences; Epitope E: WYIKIFIMIVGGLVGLRIVF, range from 90-100%, 3 out of 100 are 90%, 82 out of 100 are 95%, and 15 out of 100 are 100%); Epitope F: PEGIEEEGGERDRDTRGRLV, range from 85-100%, 8 sequences are 85%, and 92 out of 100 are 100%, and Epitope G: IWVDLRSLFLFSYH, range from 92.9-100%, 2 out of 100 are 92.9 and the rest of them are 100%. Some peptides demonstrated binding to sera only at low dilution, while some maintained high binding ability even at higher dilutions.

The following are the peptides (designated by NIH AIDS Research & Reference Reagent Program in 2004) to which patient LTNP sera demonstrated high binding at dilution of 1:500: 2015 (TKAKRRVVQREKRAAIGALF ) (2), 2018 (SVTLTVQARL) (4), 2019 (QARLLLSGIVQQQNNLLRAI) (5), 2020 (QQQNNLLRAIEAQQHMLQLT) (2), 2022 (VWGIKQLQARVLAVERYLKD) (5), 2025 (GKLICTTTVPWNASWSNKSL ) (4), 2031 (LLELDKWASLWNWFDITNWL) (2), 2032 (DITNWLWYIKI) (2), 2033 (5), 2038 (PEGIEEEGGERDRDTSGRLV) (4), 2039 (RDRDTSGRLVHGFLAIIWVD) (2), 2040 (IWVDLRSLFLFSYH) (4), 2042 (HRDLLLIAARIVELLGRRGW) (3), 2043 (IVELLGRRGWEVLKYWWNLL) (3), and 2046 (SLLNATAIAVAEGTDR) (2). The following peptides bound to LTNP sera at 1:2000 dilutions: 2019 (4), 2025 (3), 2031 (3), 2032 (2), 2033 (3), 2038 (2), 2040 (3), and 2047 (AVAEGTDRVIEVLQRAGRAI) (2).

**Figure 2:**
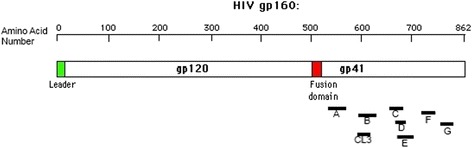
Schematic of gp160 of HIV-1 MN and epitope mapping results. The results for epitope A-G are shown in the figure accompanying the structure for the glycoprotein 160. The location and degree of overlap are shown in the figure also. The complementary information on this figure is provided in Table 1.

When mapping the LTNP samples, containing antibodies that bound with high affinity at low sera concentration, we found that there was high protein homology between them, representative of sequence conservation. These results are presented in Figure 2 and Table 1.

**Table 1:** **Summary of found epitopes from the study from LTNP sera**

**Epitope**	**Epitope sequence**	**Location in gp160**	**Sequence match**
A	QARLLLSGIVQQQMNLLRAT	Start: AA 541	100% identical (100 out of 100)
		End: AA 560	
B	GCSGKLICTTTVPWNASWSNKSL	Start: AA 598	95.7-100% identical (20 of 100, 100%; 75 of 100, 95.7%); core epitope KLIC is 100%
		End: AA 620	
C	LLELDKWASLWNWFDITNWLW	Start: AA 661	100% identical (100 out of 100)
		End: AA 681	
D	DITNWLWYIKI	Start: AA 675	100% identical (100 out of 100)
		End: AA 685	
E	WYIKIFIMIVGGLVGLRIVF	Start: AA 681	90-100% identical (3 of 100, 90%; 82 of 100, 95%; 15 of 100, 100%)
		End: AA 700	
F	PEGIEEEGGERDRDTRGRLV	Start: AA 731	85-100% identical (8 of 100, 85%; 92 of 100, 100%)
		End: AA 750	
G	IWVDLRSLFLFSYH	Start: AA 757	92.9-100% identical (2 of 100, 92.9%; 98 of 100, 100%)
		End: AA 770	
CL3	GCSGKLICTT	Start: AA 598	100% identical (100 out of 100)
		End: AA 607	

5 LTNP patients were assayed for 7 different epitopes and CL3 found to have a high degree of matching. The epitope, epitope sequence, epitope location within HIV-1 MN gp160, and the sequence match between the samples is given in the table.

### LTNP sera mediated inhibition of MT-2 infection cells by H9MN

**Figure 3:**
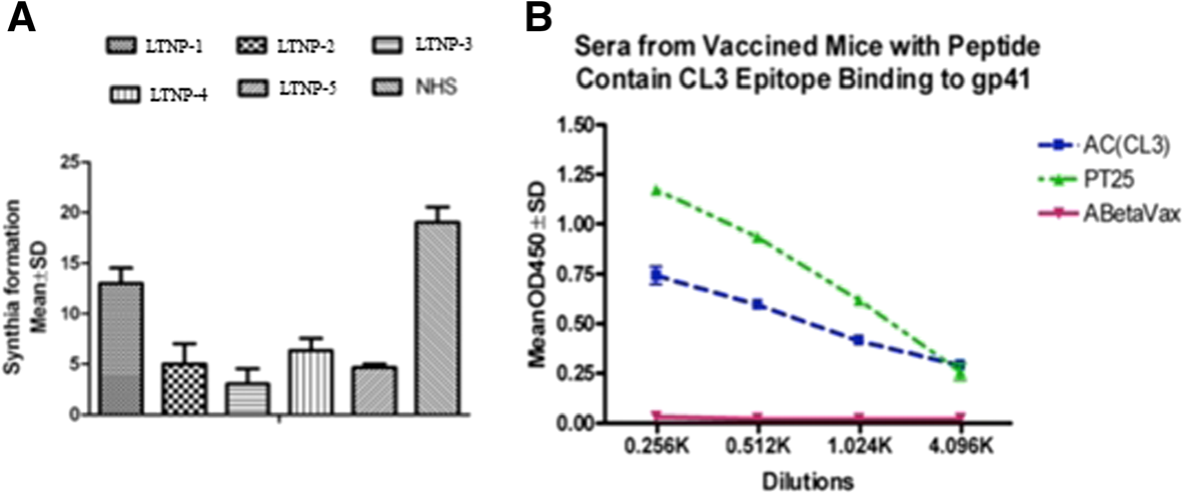
Inhibition of viral infection by LTNP sera and vaccinated mice sera. Viral infection was measured after treatment with LTNP sera and sera from mice vaccinated with a peptide containing an identified HIV neutralization epitope. **A** (left graph) demonstrated the MT2 cell syncytia formation after infection by cell free H9MN virus. As shown in the figure, sera from patient LTNP-2, LTNP-3, LTNP-4, and LTNP-5 (patient 25) significantly inhibited the syncytia formation (p < 0.05). **B** (right graph) shows antisera from the vaccinated mice (with the AC peptide) binding to gp41, and comparing this binding titer to the positive control sera of patient 25 and the negative control sera of mice vaccinated with the beta amyloid peptide.

Viral infection was inhibited both by cell-free LTNP sera and sera from mice vaccinated with CL3 chimeric peptide (containing an HIV neutralization epitope). The Figure 3A demonstrates MT2 cell syncytia formation after infection by cell-free H9MN virus. Sera from patient LTNP-2, LTNP-3, LTNP-4, and patient 25 (LTNP-5), from which the human monoclonal antibody CL3 was developed, markedly inhibited syncytia formation (p < 0.05). In Figure 3B (right graph), this shows inhibition of viral infection using antisera generated from vaccinated mice (AC peptide) binding to gp41 (Figure 3). All LTNP sera samples inhibited syncytia formation in vitro (Figure 3A). Due to the consequences, it gives credit to the idea that there is motivating interest in studying LTNP sera and the potential for finding additional neutralizing antibodies.

### CL3 neutralized HIV-1 Clade B Clinical Isolate

**Figure 4:**
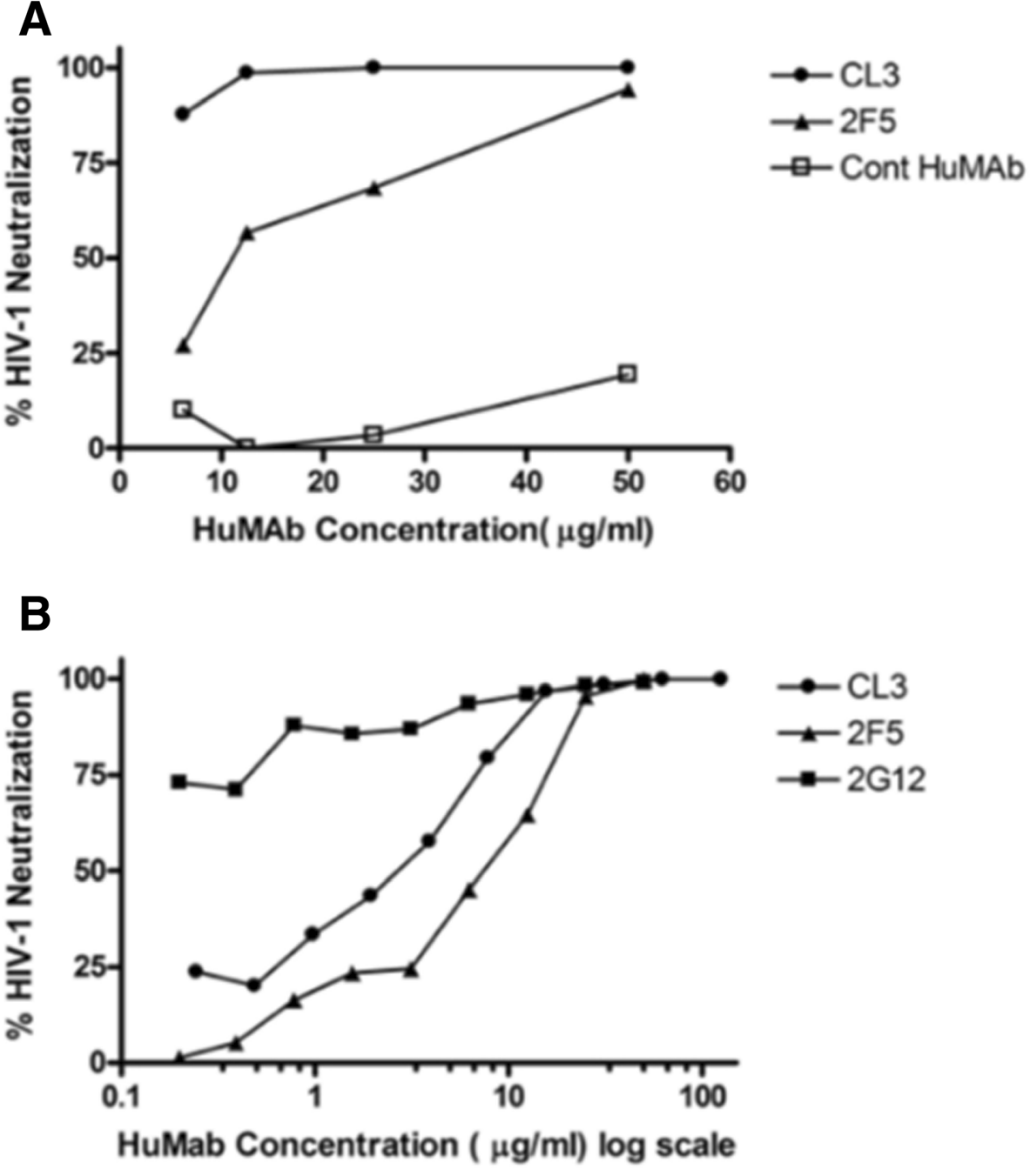
Neutralization Profiles. The neutralization profile from CL3 is provided along with results for two other established HIV neutralizing HuMabs (2F5 and 2G12). **A** CL3 and 2F5 are compared against the negative control, a control human monocolonal antibody. **B** CL3 and 2F5 are compared against the positive control, 2G12. The concentration of CL3 that inhibits 50% of cellular infections by the 92 BR030 isolate was 2 μg/ml, while the same value for 2F5 was 10 μg/ml. This indicates that, at least for this HIV-1 isolate, CL3 is more effective than 2F5 at neutralizing infection (p < 0.05).

The preparation of the monoclonal antibody has been described by Dr. Cotropia et al. [34]. The peptide GCSGKLICTTVPWNASWSNKSL, which has previously been determined to contain the HIV-neutralizing epitope KLIC of the clone 3 (CL3) antibody, was used to vaccinate mice in this experiment, and the antisera obtained from the mice were compared to the titer of the CL3 antibody. Sera from patient 25 (LTNP-5), the sera from which the human monoclonal antibody CL3 was developed, was used as a positive control. Sera from mice vaccinated with the beta-amyloid peptide served as a negative control. We generated several human monoclonal antibodies using sera from human patient 25 in our previous study, Cao et. al. (2004) [35], and we found that CL3 binds to peptide 2025. This antibody can successfully neutralize HIV-1 clade B clinical isolate (Figure 4). The neutralization profile of CL3 is provided, along with results for two other established HIV-neutralizing HuMabs (2F5 and 2G12). The concentration at which CL3 inhibits 50% of cellular infections by the 92 BR030 isolate was 2 μg/ml, while the same value for 2F5 was 10 μg/ml. This indicates that for this HIV-1 isolate, CL3 is more effective than 2 F5 at neutralizing the infection.

### CL3 and 2F5 are conserved epitopes, implying their functional importance

**Figure 5:**
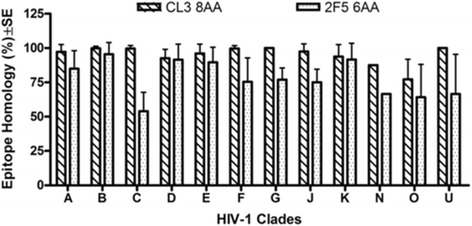
Conservation of Epitopes across HIV-1 Clades A-U. As shown above, both the CL3 and 2F5 epitopes are conserved across all HIV-1 Clades A-U. CL3 appears to have a greater percent homology across clades than the already established 2F5.

Our results show that the CL3 and 2F5 epitopes are highly conserved among different HIV-1 isolates, spanning clade A through U (Figure 5). The graph indicates the percent epitope homology among different HIV isolates across clades A through U for HuMab anti-HIV neutralizing HuMab CL3 (epitope = 8 aa long) and 2F5 (epitope = 6 aa long). The graphs indicate that CL3 consistently showed higher conservation than 2F5.

### LTNP sera show few cell mediated (cytokine) differences from NHS

**Figure 6:**
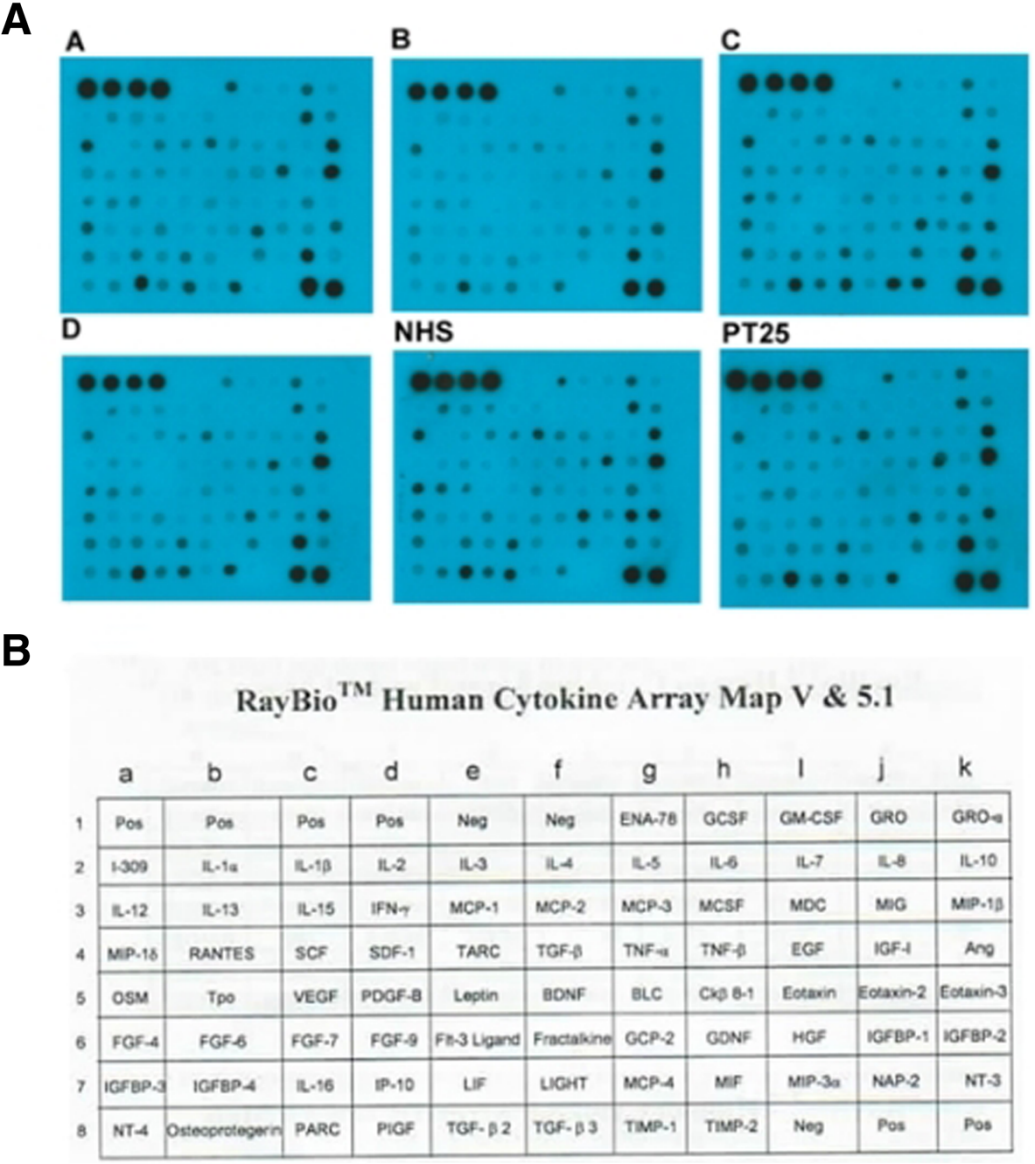
RayBio^TM^ Assay Human Cytokine Array. Select result of proteins that showed a change in expression can be found in Table 2 and manufacturers map of the array.

We conducted experiments regarding the levels of specific markers of the immune system, mainly cytokines and chemokines. Many cytokines were measured using the RayBioTM Human Cytokine Array Map V & 5.1 (Figure 6). The data from the cytokine and chemokine array showed that chemokine CCL1 (I-309) and insulin-like growth factor-1 (IGFBP1) showed significant decreases, while TIMP metallopeptidase inhibitor 2 (TIMP-2) and neutrophil-activating protein-2 (NAP-2) showed substantial increases (p < 0.05) (Figure 6, Table 2). These results suggest that both the humoral and cellular responses in the immune system of LTNP patients, mainly the LNTP sera (e.g. antibodies), may play a more significant role in preventing disease progression.

**Table 2:** **Protein changes in LTNPs samples versus normal control**

**Protein**	**Sample A**	**Sample B**	**Sample C**	**Sample D**	**PT25**
I-309 (CCL1)	−81.7%	−94.3%	−60.9%	−49.9%	−70.3%
IGFBP-1	−82.9%	−72.4%	−57.2%	−55.9%	−54.8%
TIMP-1	159.9%	44.3%	299.6%	226.2%	141.1%
NAP-2	95.6%	113.4%	142.7%	205.1%	190.1%

Percent change was calculated upon the quantification to protein array result of each LTNPs sample using the following formula: (LTNPs-normal)/normal X 100%.

## Discussion

This report presents data pertaining to the use of binding and neutralization pattern assays used on the sera of HIV-infected LTNP patients to guide the development of HIV immunotherapy. Although a fair amount of work has been carried out on this idea, there is currently no existing single or cocktail antibody preparation that is used clinically against HIV/AIDS.

In this report, we demonstrated that a LTNP serum does, in fact, contain binding and neutralization information that may contribute to the development of future therapeutic antibody cocktail formation. The mapping results, supported by the protein sequence homology analysis, demonstrated that all identified epitopes are highly conserved. More importantly, all potent neutralizing antibodies against gp41 (2F5, 4E10 and CL3) were covered in our mapping result. This procedure may be used on other components of the HIV virus to develop potent neutralizing antibodies.

The high degree of conservation among epitopes for HuMabs, such as CL3, may have success as immunotherapeutics against broadly divergent HIV-1 clades [36]. This is also backed by the cross-clade reactivity exhibited by monoclonal antibodies to the CD4 binding domain, the C-terminus of gp120, and to regions on gp41 [17,22,37,38]. Unfortunately, the vaccine developed in this study does not have an antibody as potent, in terms of neutralizing capability, as CL3, which may be due to a change in the epitope in the chimeric peptide. We are currently working to further improve the function of the chimeric vaccine, and plan to use this procedure to study other portions of the HIV-1 clinical isolate.

As indicated, anti-HIV HuMAbs against conserved regions of the gp41 envelope glycoprotein are candidates for clinical use because of their ability to broadly neutralize many clinical isolates. However, to date there are only a few HuMAbs that possess neutralizing activity. Of note, 2F5 recognizes a relatively conserved region in the ectodomain of gp41 near the transmembrane region of the molecule [39]. Others are 4E10 and Z13, which are specific for a region that is located immediately next to 2F5 epitope towards the carboxy terminal.

Interestingly, the “immunodominant domain” region, which contains the epitope for CL3, has produced other HuMAbs that have been shown not to have neutralizing capabilities [40,41]. Viveros et al. reported that the CL3 epitope is linear and exists within the immunodominant domain of gp41 but that it appears to be rarely immunogenic. This was based on an inability to produce polyclonal antibodies in rabbits against this epitope using appropriate [32]. However, the generation and biological activity of the CL3 HuMAb indicate that, under certain circumstances, this epitope is accessible, and therefore not entirely immuno-silent. This suggests that in some individuals, through mechanisms not completely understood, changes in accessibility occur during the course of infection that result in an exposure of these neutralization epitopes [36,42].

Protein array results from the cytokine assay identified that I-309 and IGFBP-1 significantly reduced in LTNPS, but TIMP-1 (tissue inhibitors of MMPs-1) and NAP-2 (neutrophil-activating protein-2) significantly increased in those subjects. The roles of those cytokines are not quite clear, but they are all related to active T cell function. It is important to mention, however, that I-309 can inhibit X4 infection [43] and that IGFBP-1 is 100% positive in HIV infected subjects [44]. Activation of the membrane-type-1-matrix-metalloproteinase and the induction of the membrane-bound tissue inhibitor of metalloproteinase-2 (TIMP-2) can increase HIV infection [45]. NAP-2 can enhance antiviral activity of INF gamma [46]. Our result further suggests that the cellular response here may also play a critical role in the prevention of the progression of HIV to AIDS in diseased subjects.

Additional research is needed to further characterize the antibody library harbored in LTNP sera by using a similar antibody-development procedure as used in this study in finding the CL3 neutralizing antibody. In summary, the results we have reported demonstrate a potentially reliable and practicable approach towards antibody cocktail development, applicable for HIV prevention and vaccine design. We may be able to obtain the solution to deal with HIV/AIDS through the study of more LTNP subjects.

## Conclusions

Our results indicate that the use of LTNP samples may be a useful for finding antibody cocktail candidates and antiviral agents for the development of HIV/AIDS treatments. The sera of the LTNP patients were found to be able to bind to the same peptide as clone 3 (CL3), the broad neutralizing HIV-neutralizing monoclonal antibody against gp41. This procedure may be replicated on other portions of the HIV virus to develop equally potent antibodies, like CL3, in the development of neutralizing antibodies or antibody compounds. When looking further at the cytokine and chemokine changes in tracking the overall changes to the immune system, we found that there were significant changes in a handful of cytokine and cytokine markers. This suggests that both the humoral and cellular immune responses play a role in the prevention of HIV disease progression. We believe that CL3 may be clinically useful with regards to development of antibodies and vaccines against HIV due to its great potential for viral inhibition and its high binding affinity, and that further antibodies can be found from LTNP patients with similar potency using the methods presented here.
